# Detecting very low allele fraction variants using targeted DNA sequencing and a novel molecular barcode-aware variant caller

**DOI:** 10.1186/s12864-016-3425-4

**Published:** 2017-01-03

**Authors:** Chang Xu, Mohammad R. Nezami Ranjbar, Zhong Wu, John DiCarlo, Yexun Wang

**Affiliations:** Life Science Research and Foundation, Qiagen Sciences, Inc., 6951 Executive Way, Frederick, Maryland, 21703 USA

**Keywords:** Variant caller, Molecular barcode, Statistical model, PCR enrichment

## Abstract

**Background:**

Detection of DNA mutations at very low allele fractions with high accuracy will significantly improve the effectiveness of precision medicine for cancer patients. To achieve this goal through next generation sequencing, researchers need a detection method that 1) captures rare mutation-containing DNA fragments efficiently in the mix of abundant wild-type DNA; 2) sequences the DNA library extensively to deep coverage; and 3) distinguishes low level true variants from amplification and sequencing errors with high accuracy. Targeted enrichment using PCR primers provides researchers with a convenient way to achieve deep sequencing for a small, yet most relevant region using benchtop sequencers. Molecular barcoding (or indexing) provides a unique solution for reducing sequencing artifacts analytically. Although different molecular barcoding schemes have been reported in recent literature, most variant calling has been done on limited targets, using simple custom scripts. The analytical performance of barcode-aware variant calling can be significantly improved by incorporating advanced statistical models.

**Results:**

We present here a highly efficient, simple and scalable enrichment protocol that integrates molecular barcodes in multiplex PCR amplification. In addition, we developed smCounter, an open source, generic, barcode-aware variant caller based on a Bayesian probabilistic model. smCounter was optimized and benchmarked on two independent read sets with SNVs and indels at 5 and 1% allele fractions. Variants were called with very good sensitivity and specificity within coding regions.

**Conclusions:**

We demonstrated that we can accurately detect somatic mutations with allele fractions as low as 1% in coding regions using our enrichment protocol and variant caller.

**Electronic supplementary material:**

The online version of this article (doi:10.1186/s12864-016-3425-4) contains supplementary material, which is available to authorized users.

## Background

Detecting somatic mutations by next generation sequencing (NGS) is a critical part of cancer research and diagnostics. In tumor samples and circulating nucleic acids, mutations may be present in a very low fraction of DNA molecules for reasons such as normal tissue contamination or mutations occurring in a small subset of tumor cells. These very low level somatic mutations have been the target of intense investigations in recent years, because they hold great promise for early detection of cancers, monitoring the effectiveness of targeted therapies and early intervention against drug resistant clones.

In order to detect these very low allele fraction mutations, deep sequencing coverage is normally required since mutations need to be observed on a sufficient number of reads to pass predetermined variant calling threshold. For example, to observe a 2.5% variant on minimum two reads with 90% probability, a minimum of 200x coverage is required based on the binomial distribution. In practice, 1000x or higher coverage is recommended to call 2.5% variants [1]. Targeted sequencing enables researchers to focus sequence capacity on a small genomic region of interest, making it an appealing approach to achieve very deep coverage in a cost effective way. Both hybridization capture and PCR amplicon based approach have been used in enriching specific genome regions. The PCR amplicon approach is preferred by many researchers due to its low DNA input requirement, simple protocol and fast turnaround time. However, the inability to identify read duplication of the same sample fragment is an inherent limitation in typical PCR amplicon sequencing.

Having deep sequencing coverage alone is not sufficient for detecting mutations at very low allele fractions. More importantly, researchers need enough original DNA molecules sampled in the sequencing workflow. For example, detecting 1% mutations reliably from the starting materials of 100 genomes is extremely difficult, no matter how many read duplications are produced. This becomes a significant issue for damaged FFPE samples, as the optical measurement of the DNA input does not reflect the real number of usable molecules. A target enrichment workflow needs to efficiently capture a large number of original DNA molecules. Furthermore, read duplication usually confounds the estimation of the number of DNA molecules captured.

Besides read depth and DNA input, detecting mutations at very low allele fractions is challenged by errors introduced in many steps of the NGS process. In PCR-based targeted sequencing, target templates are bound with gene-specific primers and amplified via multiple PCR cycles. Given enough input, a large amount of DNA fragments will be captured so that even very low allele fraction mutations can be observed on plenty of reads. However, the reads often contain errors that are difficult to distinguish from true variants. The errors are accumulated during the critical steps of an NGS protocol, including library preparation, sequencing, and read alignment. For example, in PCR amplification, DNA polymerases have an error rate of 10^−6^ per base [2]. Sequencing errors typically occur at a higher rate. Studies have shown that Illumina MiSeq platform has an average error rate of 0.006 to 0.01 per base, depending on how far away the base is from the read start and other factors [3]. Other literature indicates that the error rate is between 0.01 to 0.1 per base on general Illumina platforms [4]. Errors can also happen to the read alignment algorithms, especially when a read fails to span a repetitive region or covers a region containing complex variants near each other. Low allele fraction and relatively high error rate together lead to poor signal-to-noise ratio, making it difficult to identify true variants without generating false positive variant calls. Moreover, because DNA fragments are not evenly amplified due to resampling bias [5, 6], the observed variant allele fractions are often skewed, which may ultimately lead to inaccurate variant calls.

Molecular barcoding technology aims to reduce the impact of enrichment and sequencing artifacts and has the potential to improve mutation detection accuracy [6–11]. In brief, each original DNA molecule is tagged with a unique molecular barcode. After amplification and sequencing, the barcode sequence can be retrieved from the reads, allowing each read to be traced back to its original DNA molecule. Molecular barcoding has recently been implemented in several NGS target enrichment protocols and researchers have demonstrated its utility in somatic variant detection and RNA quantification [8, 11]. Quantification of template molecules can be done by counting the number of unique barcodes rather than the number of total reads, which reduces PCR resampling bias and improves quantification accuracy. Variant detection can also benefit from molecular barcodes because sequencing errors can often be identified by comparing across the reads containing the same barcode.

The progress in enrichment and sequencing technology brings about more complicated NGS data that require advanced bioinformatics tools to analyze. Bayesian and other statistical models have been applied in variant calling algorithms such as SNVSniffer [12], FaSD [13], and MuTect [14]. SNVSniffer models the read counts of the observed genotypes using a multinomial distribution, and infers the true genotype that has the largest posterior probability calculated under a Bayesian framework. MuTect evaluates the likelihood of variant using the log odds score, which is the likelihood ratio of mutation versus wild type. FaSD is a germline variant caller that evaluates the SNP probability by calculating an alternative score from a binomial distribution-based model. However, these variant callers were developed for sequencing data without molecular barcode. Appropriate analytical methods are needed to take full advantage of the molecular barcode information. A two-step approach has been proposed and has demonstrated utility in detecting variants as low as 1% [8, 15]. At first, a consensus sequence is constructed for each barcode. Next, downstream analyses (include variant calling) are performed on the set of consensus sequences with existing bioinformatics tools such as the aformentioned ones. There are several downsides to this approach. First, useful information may be lost when constructing the consensus sequences. For example, in VarDict, the ratio between low quality and high quality reads is an important parameter in the variant calling algorithm [16]. However, the ratio will likely be skewed in the consensus sequence set. Second, building a base quality score system for the consensus sequences that mimics the Phred scores used in the raw reads is difficult. The downstream variant caller cannot achieve optimal performance if skewed quality scores are passed to it, because the Phred base quality score is a centerpiece in most variant calling algorithms. Third, from a practical standpoint, maintaining and updating software from different sources can be time consuming. For example, many trial-and-error iterations might be required to tune the parameters in both the consensus-generating algorithm and the third party variant caller to make them compatible. We believe that a full-fledged variant caller that integrates molecular barcode information into the statistical algorithm will be valuable to researchers in the field.

We have developed smCounter, a barcode-aware variant caller that detects single nucleotide variants (SNV) and short insertion and deletions (indel) at very low allele fraction. smCounter applies a Bayesian probabilistic model to evaluate the read evidence for each nucleotide as well as possible indels at a target position, and compares the strength of evidence with a preselected threshold to make variant calls. smCounter also implements several filters to further reduce false positive calls. Using a simple protocol that integrates molecular barcodes into multiplex PCR enrichment, we have generated two targeted sequencing datasets from samples containing defined mutations at 1-5% allele fractions. smCounter has been run on both datasets and has demonstrated very good sensitivity and specificity in detecting coding region variants at 1% allele fraction.

## Results

### Multiplex PCR enrichment with molecular barcodes

**Fig. 1:**
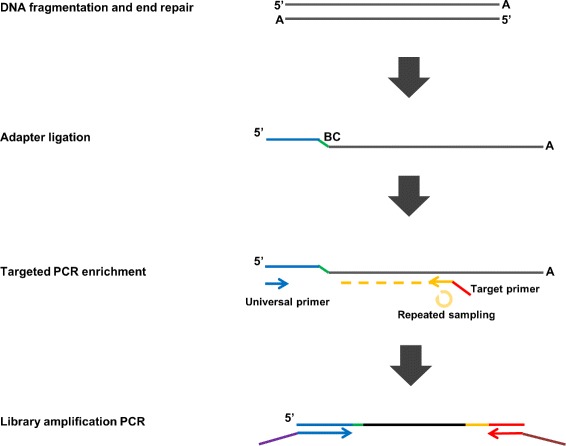
Multiplex PCR enrichment workflow with single primer

We have developed a protocol that incorporates molecular barcodes into the multiplex PCR primer enrichment process (Fig. 1). Briefly, template DNA is enzymatically fragmented and end repaired to approximately 300-500bp. Then a modified Illumina adapter containing molecular barcodes is ligated to the 5’ end of the DNA fragments. After removing unused adapters, a few PCR cycles are conducted using an Illumina adapter primer and a pool of single primers, each carrying a gene specific sequence and a 5’ universal sequence. During this process, each single primer repeatedly samples the same target locus from different DNA templates. Afterwards, additional PCR cycles are conducted using universal primers to attach complete Illumina adapter sequences and to amplify the library to the desired quantity.

Compared to existing target enrichment approaches, our method relies on single end adapter ligation, which inherently has a much higher efficiency than requiring adapters to ligate to both ends of the dsDNA fragment. More DNA molecules will be available for the downstream PCR enrichment step. PCR enrichment efficiency using one primer is also better than conventional two primer approach, due to the absence of an efficiency constraint from a second primer. During the first PCR cycles, primers have repeated opportunities to convert (i.e. capture) maximal amount of original DNA molecules into amplicons. All three features help to increase the efficiency of capturing rare mutations in the sample. In addition, incorporated molecular barcodes in the amplicon are the key to estimating the number of DNA molecules captured and to greatly reducing sequencing errors in downstream analysis. Single primer enrichment also has the potential to discover unknown structural variants, such as gene fusions.

### Data generation

In order to develop and benchmark our barcode aware variant caller, we created DNA samples with defined variants at low allele fractions, to simulate low fraction somatic mutations in tumors. DNA from NA12878 was mixed at 10% and 2% with DNA from NA24385. The genotypes of both subjects have been extensively studied by the Genome in a Bottle Consortium (GIAB) [17, 18]. The high confidence set of NA12878’s variants and an initial set of NA24385’s variants have been released to the research community. For our variant calling purpose, we defined a “ground truth” variant set by masking the NA24385 variants from the high confidence NA12878 variants. This sample mixing approach has been adopted in several other studies for benchmarking variant callers [8, 19, 20].

A first panel (N0015) was designed for algorithm development and optimization. This panel covers variant-rich hot spots by design in order to provide maximum number of variants to optimize sensitivity (Additional file 1). The first enrichment library was prepared using 10% NA12878 admixture (resulting in 5% fraction for unique heterozygous NA12878 variants). Later we also in silico diluted NA12878 molecules to 2% to simulate 1% variants for algorithm development. The in silico dilution procedure is described in Additional file 2: Supplementary Methods. Variant allele fractions before and after the dilution are illustrated in Additional file 2: Figure S1. Although suitable for algorithm development, N0015 does not resemble a typical cancer sequencing panel because the enriched region contains many variants within short distance of others, and contains very little (less than 10% of target region) coding region. Therefore, a second gene panel (N0030) covering the coding region of 194 cancer-related genes was designed to reflect the intended application of cancer sequencing (Additional file 3). Enrichment and sequencing were done using 2% NA12878 admixture for this panel, so unique heterozygous NA12878 variants were at 1% allele fraction.

Both libraries yielded highly uniform and specific coverage of the target regions, demonstrating the superior performance of our enrichment protocol. Both sequencing runs achieved very deep coverage in terms of read depth as well as barcode depth. Detailed information on the sequencing performance can be found in Table 1.

**Table 1:** Descriptive statistics of the sequencing runs N0015 and N0030

	N0015	N0030
Target region in base pairs	395,303	681,980
% in coding region	8	100
Mean read pairs per base	42,562	34,169
Mean barcode per base	4,825	3,612
Mean read pairs per barcode	8.5	8.6
% Bases >0.2× mean read depth	96.3	99.3
% NA12878	10	2
Unique heterozygous NA12878 SNP	6,175	223
Unique heterozygous NA12878 INDEL	701	49

### Overview, development, and performance of smCounter

smCounter has two main components: a statistical model to evaluate the likelihood of a variant and several post-processing filters to further reduce false positives. At each target locus, posterior probabilities of the alleles (include possible indels) are first calculated on the barcode level, noted as *P*(Allele|BC_*k*_) for the *k* ^*th*^ barcode. Assuming the locus is covered by *N* mutually independent barcodes, a prediction index $I =-\sum _{k=1}^{N}\log _{10}(1-P(\text {Allele}|\text {BC}_{k}))$ is given to each allele, representing the likelihood that the allele exists in at least one DNA molecule. If a non-reference allele’s prediction index exceeds the preselected threshold, this allele is considered as a candidate variant. Candidate variants will be confirmed only if they pass all the post-processing filters. Details of the statistical model and filters can be found in the Methods section.

**Fig. 2:**
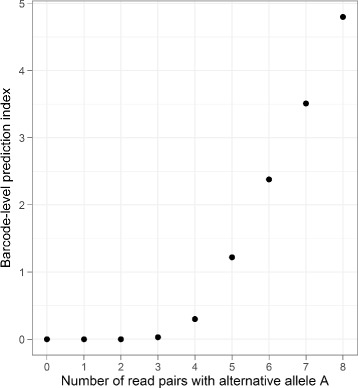
Illustration of how barcode level read evidence is evaluated for a hypothetical barcode with 8 read pairs, assuming all reads have either T (reference) or A (alternative) at the specific locus. The x-axis is the number of read pairs with the alternative allele A, ranging from 0 to 8. The y-axis is − log10(1−*P*(A|BC)), the amount of read evidence for *A* assign to the barcode. Overall, the amount of read evidence for *A* is close to 0 when less than half of the reads are *A*, and then gradually increases

The development of smCounter, including model establishment and refinement, filter design, parameter optimization, and data cleaning steps, was mainly based on over 6700 variants in panel N0015. Over 90% of N0015’s target loci are in non-coding region where repetitive sequences are much more abundant compared to typical coding regions. These challenging genomic regions provided rich training data for us to develop specific filters that significantly reduced the false positive rate in homopolymer, low complexity, and micro-satellite regions. In addition, we used the variants to refine the statistical model to take full advantage of the barcode information. In particular, we optimized the estimation of barcode level allele probability *P*(Allele|BC_*k*_) to ensure that the estimated probabilities gradually degrade to zero as the read evidence weakens (Fig. 2). We also used the barcode level allele probability to develop the “strong barcode” filter, which rejects candidate mutations lacking enough barcodes with good read evidence. smCounter achieved good overall performance in calling 5 and 1% SNVs, showing higher sensitivity in coding region compared to non-coding region (Additional file 2: Figure S2 and Additional file 2: Table S1).

**Fig. 3:**
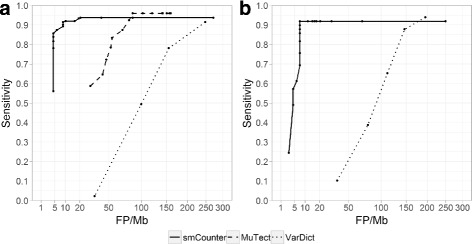
Variant calling performance on 1% variants in panel N0030. X-axis is the number of false positives per megabase and y-axis is sensitivity. *Solid lines*, *dashed lines*, and *dotted lines* represent smCounter, MuTect, and VarDict respectively. Each point on the ROC curve represents a threshold value. **a** ROC curves of smCounter, MuTect, and VarDict base on 223 SNVs. **b** ROC curves of smCounter and VarDict on 49 indels. Note that MuTect does not call indels

Given the good results from N0015, we then ran smCounter on the cancer-related gene panel N0030 and benchmarked its performance against the GIAB ground truth set using RTG tools [21]. We also ran MuTect [14] and VarDict [16] for this panel to assess the variant calling performance under our enrichment protocol without using molecular barcode information (Additional file 2: Supplementary Methods). The receiver operating characteristic (ROC) curves for all three variant callers on N0030 are presented in Fig. 3. The performance metrics (sensitivity, specificity, positive predictive value) under the optimal cutoff, heuristically defined as the point on the upper-left corner of the ROC curve, are listed in Table 2. The performance data from N0030 demonstrate that smCounter is able to detect 1% SNVs at over 90% sensitivity with less than 20 false positives per megabase of the target coding region. The performance data also show, with a lower confidence level due to small sample size, that smCounter can detect 1% indels at over 90% sensitivity with less than 10 false positives per megabase in the coding region. Overall, smCounter achieved comparably high sensitivity as MuTect or VarDict but with much fewer false positives, demonstrating the value of molecular barcoding for low fraction variant calling.

**Table 2:** Performance of smCounter, MuTect, and VarDict in detecting 1% variants in the coding region of N0030. Cutoffs were selected to represent optimal performance

Variant type	Variant Caller	TP	FP	FN	TPR(%)	FP /Mb	PPV(%)
SNV	smCounter	205	7	18	91.9	10	96.7
	MuTect	214	58	9	96.0	85	78.7
	VarDict	204	169	19	91.5	248	54.7
INDEL	smCounter	45	5	4	91.8	7	90.0
	VarDict	43	100	6	87.8	147	30.1

### Variant calling on reduced barcode and read depth

**Fig. 4:**
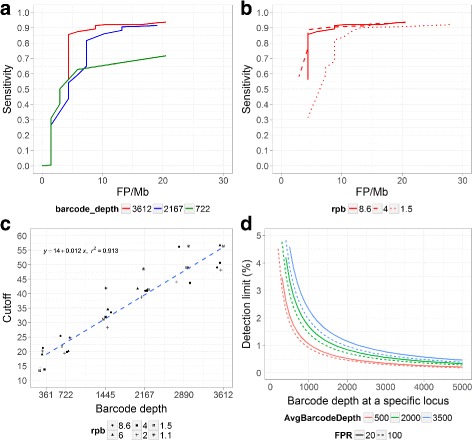
smCounter performance on calling 1% SNVs in N0030, based on reduced barcode depth and rpb. For better visualization, only a subset of barcode depth and rpb combinations are plotted. Cutoff selection and detection limit estimation are also based on the same downsampled data. **a** ROC curves at barcode depth of 3,612 (full data), 2,167, and 722 with rpb fixed at 8.6. **b** ROC curves at rpb of 8.6 (full data), 4.0, and 1.5 with barcode depth fixed at 3,612. **c** Optimal cutoffs that give the maximum sensitivity while allowing no more than 20 FP/Mb at varying barcode depth (x axis) and rpb (represented by different symbols). The *dashed line* is the linear regression equation of cutoff versus barcode depth across all rpb values. **d** Estimated detection limit as a function of locus-specific barcode depth, overall average barcode depth, and false positive rate (FPR) allowed. The y-axis is the lowest variant allele fraction that can be detected with at least 95% probability based on a binomial distribution

In practice, variant calling accuracy may be limited by the amount of sample input and sequencing capacity. To characterize smCounter’s performance under imperfect sequencing conditions, we downsampled the N0030 read set to 80, 60, 40, 20 and 10% of barcode depth (i.e., average number of barcodes per base) and the read depth to 6.0, 4.0, 2.0, 1.5, 1.1 per barcode. The performance of smCounter at all barcode depth and read pairs per barcode (rpb) combinations are demonstrated in Additional file 2: Figure S3 and S4. SNV calling results in a subset of this parameter space are highlighted in Fig. 4. We observe that smCounter’s performance degrades gradually as the barcode depth and rpb decrease. We also observe that 4 read pairs (or 8 reads) per barcode is sufficient in most cases and additional read replications provide little additional value. The downsampling procedure is described in the Additional file 2: Supplementary Methods.

### Cutoff selection

Selecting the right cutoff is critical to achieving good performance for most variant callers. However, it is often unclear to users on how to choose the cutoff value, partly because the optimal cutoff usually correlates with the unknown amount of DNA molecules captured. With molecular barcodes, we are able to provide users with a guideline on cutoff selection based on the barcode depth and the desired false positive rate. To illustrate the method, we define the optimal cutoff value as the one that gives the maximum sensitivity while allowing no more than 20 false positives per megabase. Using the downsampled N0030 data, we obtained the optimal cutoff values of smCounter at all barcode depth and rpb combinations. Based on both visual inspection (Fig. 4) and a one-way ANOVA analysis, the optimal cutoffs do not differ significantly in different rpb groups (F-test *p*=0.901). Pooling all rpbs together, the optimal cutoff value has a strong positive linear relationship with the barcode depth (*r* ^2^=0.913, F-test *p*=2.5×10^−16^). We propose a simple linear equation *y*=14+0.012*x* to predict a recommended cutoff value, where *y* is the recommended cutoff value and *x* is barcode depth. We also provide optimal cutoff values for other desired false positive rates, as shown in Table 3.

**Table 3:** Estimated optimal cutoffs based on various false positive rates (FPR) at 20, 50, and 100 per megabase and barcode depths from 500 to 3,500. The prediction equations at each FPR is also given

FPR (Mb^−1^)	Prediction equation	500	1000	1500	2000	2500	3000	3500
20	*y*=14+0.012*x*	20	26	32	38	44	50	56
50	*y*=15+0.0092*x*	20	25	29	34	38	43	48
100	*y*=13+0.0088*x*	18	22	27	31	35	40	44

### Variant detection limit

**Fig. 5:**
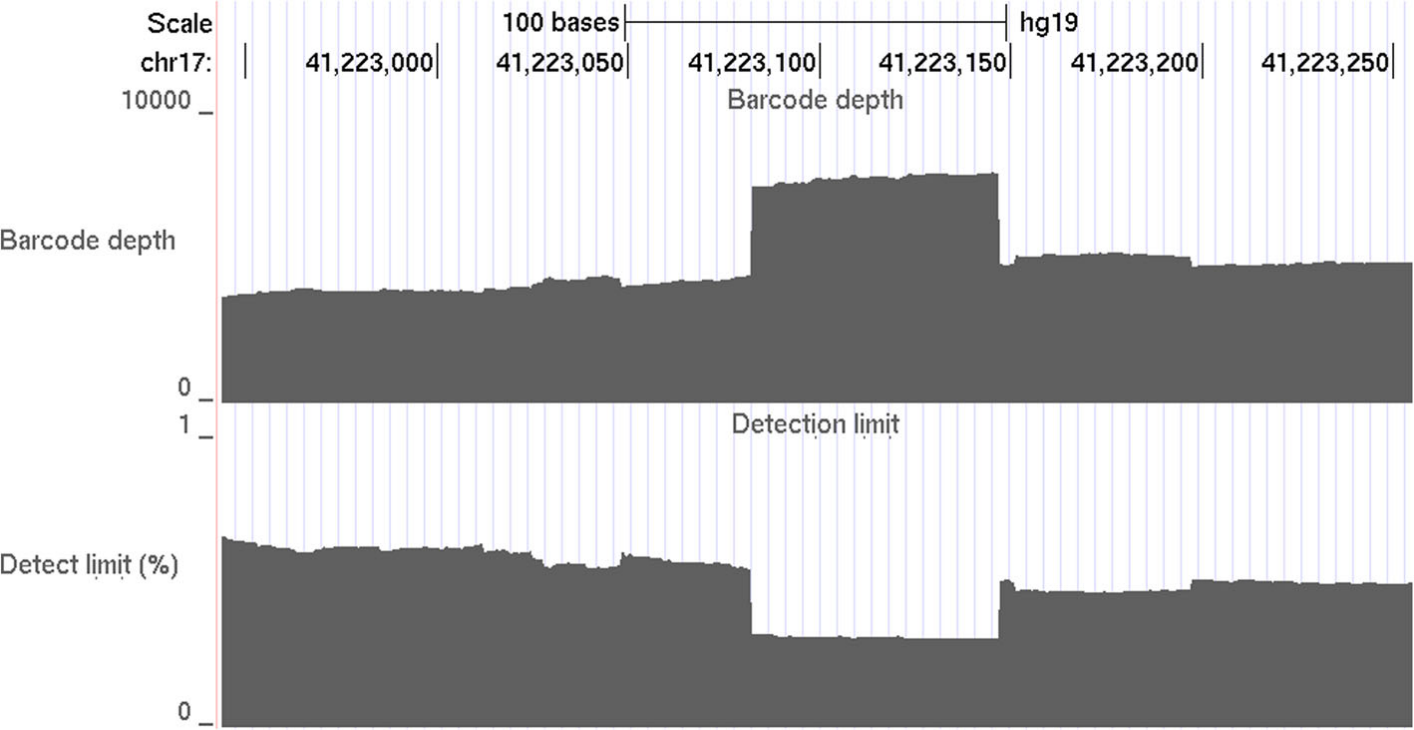
An example of barcode and detection limit map on BRCA1 exon chr17:41222944-41223255 based on N0030. The maps were generated using UCSC Genome Browser

Molecular barcodes allow us to estimate the locus specific detection limit, defined as the lowest variant allele fraction that will be called. The detection limit is mainly determined by the barcode depth at the locus, the average barcode depth of the sequencing run, and the false positive rate allowed. Within a target region, loci with higher barcode depth will have lower detection limits (more detection power), simply because the likelihood of observing real low fraction variants is higher. On the other hand, when comparing two different sequencing runs, a locus with the same barcode depth in both runs will have higher detection limit (less detection power) in the read set with higher average barcode depth, because a more stringent cutoff value will be used to control the false positive rate, as proposed earlier. Finally, the more false positives one allows, the lower detection limit one can achieve. The estimation of locus specific detection limit is illustrated in Fig. 4 and explained in details in the Methods section. Similar to a coverage map, a detection limit map can be built for a region to provide a visual inspection tool (Fig. 5). Knowing this information is particularly important in guiding the proper interpretation of negative variant calls in FFPE samples. A desired detection limit may be achieved by simply increasing the DNA input based on observed molecular barcode counts.

## Discussion

In this paper we presented a simple PCR based target enrichment protocol that integrates molecular barcodes in the sequencing reads. We demonstrated that our protocol can enrich a large genome region with high efficiency, specificity, and uniformity. The presence of molecular barcodes in the reads provides us with an effective means to estimate the number of unique DNA molecules sequenced and to effectively distinguish true variants at very low fractions from sequencing errors in the reads. Using our enrichment protocol and reference materials from the Genome in a Bottle consortium, we generated datasets containing defined DNA variants at very low allele fractions. Using this data, we developed our barcode aware variant caller and evaluated its performance.

smCounter is the first full-fledged variant caller that integrates molecular barcode information into a statistical model and has demonstrated very good sensitivity and specificity in detecting 1% SNVs and indels within targeted coding regions. Still, we acknowledge several limitations of smCounter as well as the enrichment technology.

During the development of smCounter using N0015 dataset, we observed decreased variant calling performance in non-coding regions compared to coding regions. In particular, the current version of smCounter has reduced power in detecting low allele fraction indels in non-coding regions (Additional file 2: Figure S2). The decreased performance is mainly due to more repetitive sequences and additional nearby variants (e.g. indel followed by nearby SNP) within non-coding regions of N0015 that caused read alignment errors. Our design strategy for N0015 panel to maximize the number of variants covered within a small target region likely resulted in over-representation of challenging genomic regions such as homopolymers, simple repeats, and low complexity regions (Methods). Many of the true variants within or flanked by these regions are falsely rejected by our stringent filters. Tuning the filter parameters towards more leniency will lead to higher sensitivity but also more false positives. A more practical approach is to train highly specific filters based on large amount of variants in the challenging regions using statistical or machine learning methods. Fundamentally, higher variant calling accuracy in the challenging regions should be achieved by improving the read alignment quality. One promising advance in this field is to use graph-based genome representation for read alignment instead of commonly used linear representation of the reference genome [22].

Like other variant callers, smCounter’s performance is largely determined by the preselected threshold and the optimal threshold is an unknown function of numerous factors, including sample input, DNA quality, sequencing depth, sequencing platform, etc. We have observed a linear relationship between the optimal threshold and the barcode depth and provided an empirical formula to predict the optimal threshold for a range of barcode depths and rpb levels. The robustness of the formula under the conditions outside the tested range is unclear. Moreover, the predictive formula may need to be empirically modified under significantly different PCR chemistry or sequencing error rate due to platform change.

In the enrichment protocol we used, a complete barcoded amplicon is only formed after the first target specific primer extension event. In principle, any polymerase error introduced in this very first step cannot be distinguished from real variants based on individual barcode consensus. So variant detection is theoretically limited by the polymerase fidelity during the first PCR enrichment cycle. In fact, we have observed several false positive calls that cannot be easily explained by obvious reasons and were possibly due to early polymerase errors. In practice, we have leveraged additional information (strand bias filter) to reduce this type of false positives. For example, two primers can be designed to target the locus in both sense and anti-sense orientations. The chance is extremely low that polymerase error happens at the same locus during primer extension events on both strands. Thus, many false positives can be eliminated based on biased distributions between sense and anti-sense strand. A more elegant approach is to employ duplex sequencing [7] by directly pairing the sense and antisense strand information at the single molecule level rather than at the population level. We are currently working on a scheme to integrate duplex sequencing into primer enrichment step, and we plan to report our results in a future publication.

Our current enrichment protocol also has limited ability to distinguish DNA damage induced artifacts from real variants. Artifacts induced by base damage could be common in FFPE samples depending on how the samples are prepared [23]. To identify such artifacts, one must examine both strands of DNA, because the damage to both stands is usually not identical. Similar to dealing with early PCR errors, we can use biased distributions between sense and anti-sense strand to eliminate some artifacts. A better direction is to employ duplex sequencing.

Accurate detection of very low fraction variants in DNA holds big potential in our understanding of tumor heterogeneity and in early disease diagnostics. We believe our improved enrichment protocol, benchmarking dataset and barcode-aware variant caller will provide valuable resources for continued progress by the research community.

## Conclusions

We developed a targeted DNA enrichment protocol that integrates molecular barcodes into original DNA molecules. We also developed smCounter, a somatic variant caller that integrates the molecular barcode information into a Bayesian probabilistic model. We have demonstrated that, with our protocol and variant calling algorithm, we can detect 1% allele fraction variants in coding region with over 90% sensitivity and less than 20 false positives per megabase. We believe our method will be an important contribution to cancer sequencing and somatic mutation detection.

## Methods

### Preparation of in vitro sample mixtures

Human genomic DNA samples of NA12878 and NA24385 were purchased from Coriell Institute for Medical Research (Camden, NJ, USA). Samples were mixed based on OD measurements, resulting in 10 and 2% of NA12878 DNA in the background of NA24385 DNA.

### Custom panel design

The first panel (N0015) was designed to maximize the number of unique NA12878 variants enriched by a limited number of primers. The regions were randomly selected from the genome so that two or more variants would appear within 150bp from the targeting primer. In total, 3587 primers were selected using custom scripts to balance coverage, primer Tm, dimer potential and predicted specificity within the human genome. The second panel (N0030) was designed to cover the full coding region of 194 cancer and inherited diseases related genes. 10,857 primers were selected so that 150bp or longer amplicons from those primers can overlap the entire coding region. Additional universal sequence was added to the 5’ end of all primer sequences and all oligos were synthesized by IDT (Coralville, IA).

### Single primer enrichment protocol

DNA libraries were prepared using components from QIAseq Targeted DNA Panel Kit (QIAGEN, Germany). Briefly, 80 ng DNA was enzymatically fragmented and end repaired in a 25ul reaction containing a 2.5ul 10X fragmentation buffer and a 5ul fragmentation enzyme mix. The reaction was carried out at 4 °C for 1 min, 32 °C for 24 min and 65 °C for 30 min. Immediately after the reaction, 10ul 5X ligation buffer, 5ul DNA ligase, 2.8ul 25uM barcoded adapters and water were added to 50ul. The reaction continued at 20 °C for 15 min. To ensure complete removal of free barcoded adapters, each reaction was purified for two rounds using 1.8X and 0.9X Ampure beads (Beckman, US). The purified DNA was then mixed in 20ul with 10nM each target primer, 400nM IL-Forward primer, 1X TEPCR buffer and 0.8ul HotStarTaq DNA polymerase. The PCR enrichment condition was: 95 °C for 13 min, 98 °C for 2 min; six cycles of 98 °C for 15 s and 65 °C for 15 min; 72 °C for 5 min. Each reaction was cleaned once using 0.9X Ampure beads (Beckman, US) to remove unused primers. Enriched DNA was combined with 400nM IL-Universal primer, 400nM IL-Index primer, 1X UPCR buffer and 1ul HotStarTaq DNA polymerase in a volume of 20 ul. The universal PCR condition was: 95 °C for 13 min, 98 °C for 2 min; 20 cycles of 98 °C for 15 s and 60 °C for 2 min; 72 °C for 5 min. The DNA library was purified once using 0.9X Ampure beads and quantified using QIAseq Library Quant System (QIAGEN, Germany). Both libraries were sequenced on Illumina NextSeq (pair-end, 2x150 bp) following manufacturer’s user manual (Illumina, CA).

### Read processing

Original reads in the FASTQ file are processed in the order of adapter trimming, read aligning, barcode clustering, and primer region trimming (detailed pipeline described in Additional file 2: Supplementary Methods). As a first step, non-genomic sequences such as sequencing adapters and molecular barcode region (Additional file 2: Figure S5) are trimmed off by Cutadapt [24] and custom scripts. The trimmed reads are then aligned to the reference genome using BWA-MEM [25], creating the alignment file in BAM format. A custom barcode clustering algorithm is applied to account for possible sequencing or PCR errors in the barcode sequence. In brief, the algorithm identifies barcodes that are within short edit distance (typically ≤ 1), and then combines them if one barcode has significantly more supporting reads than others. The procedure is described in more detail in [8].

Standard GATK workflow steps such as indel realignment and base quality score recalibration [26] are not implemented in this work because they are very time consuming for ultra-deep read sets. However these procedures are recommended, and can be implemented within a reasonable time for shallower reads.

### smCounter workflow

smCounter processes individual target locus sequentially and independently. At a target locus, reads covering the locus are collected by samtools mpileup and screened by several pre-processing filters. High quality reads that pass the pre-processing filters are put into the statistical model to determine if a potential (candidate) variant exists. Finally, a variant call will be made if the candidate variant passes a number of post-processing filters. We described the statistical model below and the filters in Additional file 2: Table S2, S3. In particular, we gave a detailed discussion on the strand bias filter in Additional file 2: Supplementary Methods.

smCounter follows two steps to evaluate the likelihood of mutation at a target locus. The first step aims to estimate the posterior probability of each allele using all reads originating from an individual barcode. For example, the posterior probability of an ‘*A*’ nucleotide for the *k* ^*th*^ barcode can be calculated by Bayes rule as

1 $$  P(A\mid BC_{k}) = \frac{P(BC_{k} \mid A)P(A) }{\sum_{X \in \Theta} P(BC_{k} \mid X) P(X)},  $$

where *P*(*X*) is the prior probability of allele *X*, *P*(*B* *C*_*k*_∣*X*) is the likelihood of all alleles in barcode *k* given the true allele *X* in the corresponding DNA molecule, and *Θ* includes the set of *A*,*T*,*G*,*C* and any indels observed on the reads covering the position. For simplicity, each allele is given an equal prior probability, i.e., *P*(*X*)=1/|*Θ*|, where |*Θ*| being the cardinality of *Θ*.

*P*(*B* *C*_*k*_|*X*) is a key component of the model. However, its analytical form cannot be easily obtained, so instead we give a heuristic approximation. The main assumption is that the non-*X* alleles are caused by a mixture of base-calling errors during sequencing and PCR enzymatic errors during DNA enrichment. Without knowing the relative fraction of the two errors, we approximate *P*(*B* *C*_*k*_|*X*) by the weighted sum of *P*(base-calling error) and *P*(PCR error). The idea is illustrated as follows.

Suppose we want to calculate *P*(*B* *C*_*k*_|*A*). Assume the barcode has *n* supporting reads and *n*_*A*_, *n*_*T*_, *n*_*G*_, *n*_*C*_ of the reads has *A*,*T*,*G*,*C* at the position. For simplicity, we assume there is no indel or ambiguous base, so *n*_*A*_+*n*_*T*_+*n*_*G*_+*n*_*C*_=*n*. Furthermore, we assume the Phred quality scores of ‘*A*’ bases are $q_{(A)1}, \ldots, q_{(A)n_{A}}\phantom {\dot {i}\!}$. The base-calling error probabilities are $e_{(A)1}, \ldots, e_{(A)n_{A}}\phantom {\dot {i}\!}$, where $e_{(A)j}=10^{-q_{(A)j}/10}\phantom {\dot {i}\!}$. The same notations apply to *G*,*T*,*C* reads. Given true allele *A*, the probability that *G*,*T*,*C* alleles are base-calling errors is $C_{p}\prod _{Y\in \Theta } \prod _{i=1}^{n_{Y}} e_{(Y)i}^{1-I(Y=A)} (1-e_{(Y)i})^{I(Y=A)}$. In this expression, *C*_*p*_=3×10^−5^ is approximately the probability of no PCR error, estimated assuming 30 PCR cycles and a 10^−6^ PCR error rate in each cycle. *Θ* is the set of {*A*,*T*,*G*,*C*}, and *I*(*Y*=*A*) equals 1 if *Y*=*A* and 0 otherwise. The probability that *G*,*T*,*C* alleles are PCR errors is approximated by the product of $\prod _{Y\in \Theta } \prod _{i=1}^{n_{Y}} (1-e_{(Y)i})$ (probability of no base-calling error) and $\min _{Y\in \{G,T,C\}} 10^{-6(0.5+n_{Y})/\sum _{Z\in \Theta }(0.5+n_{Z})}$ (heuristic approximation of PCR error). In summary, *P*(*B* *C*_*k*_|*A*) is approximated by

2 $$\begin{array}{*{20}l}  P(BC_{k}\mid A) &\approx \alpha C_{p}\prod_{Y\in \Theta} \prod_{i=1}^{n_{Y}} e_{(Y)i}^{1-I(Y=A)} (1-e_{(Y)i})^{I(Y=A)}  \\ &+ (1-\alpha) \prod\limits_{Y\in \Theta} \prod\limits_{i=1}^{n_{Y}} (1-e_{(Y)i}) \min_{Y\in\{G,T,C\}}\\&\quad 10^{-6(0.5+n_{Y})/\sum_{Z\in\Theta}(0.5+n_{Z})}, \end{array} $$

where *α*∈ [ 0,1] represents the relative weight between base-calling errors and PCR errors. The choice of *α* has no significant impact on smCounter’s performance (Additional file 2: Figure S6). Throughout this study, we have set *α*=0.5, which is mathematically equivalent to a direct sum of base-calling errors and PCR errors. *P*(*B* *C*_*k*_∣*X*) is calculated using the same formula for any other allele *X*. Combining all components and applying equation (1), we have the estimation of barcode level posterior probability of each allele.

The second step is to evaluate the total evidence of each allele at the locus. Assuming the locus is covered by *N* barcodes that are independent from each other, the probability that *A* is a true allele, i.e., at least one of the DNA copies has *A* at the locus, is given by $P(A) = 1-\prod _{k=1}^{N} \big (1-P(BC_{k}\mid A)\big)$. For computational purposes, we take the logarithm of 1−*P*(*A*) and define the prediction index of *A* as

3 $$  I(A) = -\sum_{k=1}^{N}\log_{10}\big(1-P(A|BC_{k})\big).  $$

*I*(*A*) can be interpreted as a measurement of the evidence for *A* at the locus. If *A* is a non-reference base and *I*(*A*) exceeds the preselected threshold, *A* is considered as a candidate point mutation.

### Benchmarking variant calling performance

The initial ground truth set consists of the high confidence heterozygous NA12878 variants (v2.19, ftp://ftp-trace.ncbi.nlm.nih.gov/giab/ftp/release/NA12878_HG001/NISTv2.19/) in the target region with NA24385’s variants (ftp://ftp-trace.ncbi.nlm.nih.gov/giab/ftp/data/AshkenazimTrio/analysis/NIST_CallsIn2Technologies_05182015/) being masked out. In addition, we identified one NA12878 SNP and ten NA24385 SNPs in the N0015 target region, as well as one NA24385 SNP in the N0030 target region, that were not present in the released variant sets. All twelve putative variants were visually verified in IGV and some were again verified in an independent read set with 80% NA12878 admixture as well as in other publicly available data for NA12878 and NA24385. The ground truth set was modified assuming these putative variants are real.

Variant calling performance was benchmarked against the modified ground truth set. Comparing variants identified from different variant callers is not a trivial task, because complex variants can often be represented in different forms. For this task we used the vcfeval function in RTG Tools [21], which performs complicated VCF comparison and determines whether different representations indicate the same complex variant.

### Estimation of locus specific detection limit based on molecular barcode counts

The detection limit at a specific locus can be estimated based on the barcode depth at the locus, the average barcode depth of the sequencing run, and the desired false positive rate. Let the locus specific barcode depth be *d* and the average barcode depth be *D*. Assume the desired false positive rate is 20 per megabase. To call a variant at the locus, the prediction index given by equation (3) needs to exceed the cutoff value approximated by 14+0.012*d* (Table 3). Let *I* be the average prediction index of a barcode with good read evidence. In N0030, *I* is typically around 3.5, which was the value used in Fig. 4. The minimum number of barcodes with the alternative allele (alternative barcode) is estimated by the ceiling of (14+0.012*d*)/*I*.

We seek the minimum allele fraction *f* such that the minimum number of alternative barcode is reached with at least 95% probability, assuming the actual number of alternative barcodes follows a binomial distribution Bin(*D*,*f*). The probability of observing sufficient alternative barcodes can then be calculated by *P*_*D*,*f*_(*X*≥(14+0.012*d*)/*I*), where *P*_*D*,*f*_ is the probability distribution function of the binomial distribution Bin(*D*,*f*). Finally, we find the detection limit *f* ^∗^ that ensures this probability is at least 95%:

$$f^{*} = \min_{f\in[0,1]} P_{D,f} \left(X \ge \frac{14+0.012d}{I}\right) \ge 0.95. $$

In this estimation, a different equation for the cutoff value or a different barcode level prediction index *I* may be used, depending on the desired false positive rate and the average number of rpb.

## Additional files

Additional file 1 The spreadsheet lists all unique heterozygous NA12878 variants in the N0015 target region. (XLSX 1136 kb)

Additional file 2 The supplementary materials include supplementary methods, supplementary figures, and supplementary tables. (PDF 706 kb)

Additional file 3 The spreadsheet lists all unique heterozygous NA12878 variants in the N0030 target region, as well as the corresponding gene names. (XLSX 67.6 kb)
