# Supplementary material for: Detecting very low allele fraction variants using targeted DNA sequencing and a novel molecular barcode-aware variant caller
Source: BMC Genomics. 2017 Jan 3;18:5. doi: 10.1186/s12864-016-3425-4 (PMC5209917; doi:10.1186/s12864-016-3425-4)
Supplement: Additional file 2 — The supplementary materials include supplementary methods, supplementary figures, and supplementary tables. (PDF 706 kb) [file 12864_2016_3425_MOESM2_ESM.pdf]

Supplementary Materials for: Detecting very low allele fraction variants using targeted DNA sequencing and a novel molecular barcode-aware variant caller

Chang Xu Mohammad R Nezami Ranjbar Zhong Wu John DiCarlo
Yexun Wang

Supplementary Methods

Diluting N0015 variants to 1% allele fraction

The DNA sample sequenced using the N0015 panel contained 5% unique heterozygous NA12878 variants. To develop the variant caller, we *in silico* diluted these variants to 1% allele fraction by downsampling NA12878 barcodes at the variant loci.

First, we obtained the genome coordinates of unique NA12878 variants by intersecting the GIAB NA12878 high confidence variant list (NA24385 variants are excluded) and our target region. The downsampling was only performed at these loci.

At each locus, we collected all the molecular barcodes whose reads cover the position. For each barcode, we calculated the probability of the reference allele $P(\text{Ref}|\text{BC})$ and the alternative allele $P(\text{Alt}|\text{BC})$ using the method described in the Methods section. A barcode covering only one variant locus is determined to originate from the background sample NA24385 if $P(\text{Ref}|\text{BC}) \geq P(\text{Alt}|\text{BC})$, and from NA12878 if $P(\text{Ref}|\text{BC}) < P(\text{Alt}|\text{BC})$. For barcodes covering at least two nearby variant loci, which is abundant in N0015 since two or more variants would appear within 150 bp from the targeting primer, we classify them by comparing the sums of $-\log_{10}(1 - P(\text{Ref}|\text{BC}))$ and $-\log_{10}(1 - P(\text{Alt}|\text{BC}))$ over all the variant loci.

Once all variant-covering barcodes classified as either NA12878 or NA24385, we randomly dropped 82% of NA12878 barcodes, so about 2% of the remaining barcodes originated from NA12878. Therefore, the downsampled N0015 read set contains mostly 1% unique heterozygous NA12878 variants, as illustrated in Supplementary Figure S1.

Downsampling N0030 to simulate reduced barcode depth and read pairs per barcode

We randomly selected 10%, 20%, 40%, 60%, and 80% of the barcodes in N0030 to simulate reduced DNA input. The barcode downsampling was done by a simple random sample across all unique barcodes. At each barcode depth (including the original 100% barcodes), we further downsampled the read depth to 6.0, 4.0, 2.0, 1.5, 1.1 read pairs per barcode (rpb) without changing the barcode counts. In specific, we first identified all barcodes in N0030 and the reads under each barcode. The barcodes with rpb = 1 were automatically selected. For barcodes with rpb ≥ 2 (single reads without mate are considered as a read pair during downsampling), the first read pair was also selected. The remaining read pairs from all multi-reads barcodes were pooled together, from which a simple random sample was drawn with probability

$$p = \frac{(b_1 + b_m)(d - 1)}{r_m - b_m},$$

where d is the desired rpb, b_1 and b_m are the number of barcodes with rpb = 1 and rpb ≥ 2, r_m is the total number of read pairs among all barcodes with rpb ≥ 2. It can be verified by arithmetic that the resulting average rpb is d.

The complete variant calling performance under all combinations of barcode depth and rpb can be found in Supplementary Figure S3 and S4.

Impact of weight parameter in smCounter

Given a true allele X, smCounter assumes that the non-X alleles originate from a mixture of base-calling errors during sequencing and PCR enzymatic errors during DNA enrichment. The likelihood of all alleles in barcode k given true allele X is estimated by a weighted average:

$$P(BC_k|X) = \alpha P(\text{non-X alleles due to base-calling errors}) + (1-\alpha)P(\text{non-X alleles due to PCR errors}),$$

where α is a weight parameter between 0 and 1. The complete formula for estimating each type of errors is given in Equation (2) in the main text. To investigate the weight impact, we set α to vary from $0.0, 0.1, 0.2, \ldots, 1.0$ and evaluated the variant calling performance on N0030 data under each α value. The results indicate that smCounter's performance is not sensitive to the choice of α (Supplementary Figure S6). The weight impact is largely canceled out when calculating the posterior probability $P(X|BC_k)$ using Bayes rule in Equation (1), main text.

Strand bias filter

The strand bias filter is designed to catch false positives that are only or mostly observed in one strand. Specifically, smCounter calculates the strand bias odds ratio by

$$\text{OR} = \frac{\text{Alternative bases in forward strand/Alternative bases in reverse strand}}{\text{Reference bases in forward strand/Reference bases in reverse strand}}.$$

To account for the bias on both strands, we define $\text{OR}^* = \max(\text{OR}, \text{OR}^{-1})$. In addition, smCounter also calculates the p-value of a Fisher's exact test. The default criterion for strand bias is $\text{OR}^* > 50$ or p-value $< 10^{-5}$. In N0030 data, the strand bias filter was triggered on 363 target loci and none of these loci has a true mutation, indicating that the default criterion is well tuned. It is worth clarifying that these numbers do not indicate that the strand bias filter prevented 363 false positive calls. In fact, only one of these loci's prediction index exceeded the cutoff and would have been falsely called without the filter.

To investigate how sensitive the strand bias filter is to the criterion, we set the odds ratio criterion to $5, 10, 20, 30, 40, 50, 60, 70, 80, 90, 100, 150, 200$ and the p-value criterion to $10^{-1}, 10^{-2}, \ldots, 10^{-7}$. The p-value had no impact on the filter performance within the range of $[10^{-7}, 10^{-1}]$. The odds ratio affected how many loci the filter rejects. However, since most of the rejected loci did not meet the prediction index cutoff and many of the loci were also rejected by other filters, the overall variant calling performance only changed slightly across the odds ratio range. The most sensitive setting (odds ratio = 5) resulted in only one more false positive indel and one less false negative SNV compared to the most conservative setting (odds ratio = 200).

Detailed description of read processing pipeline

1. Read trimming

   a. Trim 3' end of R1 reads, including 11 bp universal, 12 bp barcode, and any R2 sequencing adapter not trimmed by ILMN software.

   b. Trim 3' end of R2 read, including 21 bp universal PCR adapter and any other portion of the R1 sequencing adapter not trimmed by ILMN software.

   c. Extract first 12 bases off R2 reads as molecular barcodes, and append barcode information to the read ID.

   d. Remove the next 11 base common sequence from R2 reads.

   e. Discard read pairs when trimmed R1 or R2 reads are too short (<40 nt). They are mostly remaining primer-dimer artifacts from the enrichment reaction and bead cleanup steps.

2. Read alignment (BWA-MEM 0.7.9a-r786)
   `bwa mem ref_genome.fasta reads_R1.fastq reads_R2.fastq`

3. Drop secondary (256bit) and supplementary (2048bit) alignments, and convert to BAM
   `samtools view -Sb -F 2304`

4. Post alignment filtering

   a. Discard read pairs from .bam with the following features

      i. Either R1 or R2 read is not mapped.

      ii. Corresponding R1 and R2 reads are not mapped to the same chromosome.

      iii. Corresponding R1 and R2 reads are not mapped to the same locus (within 2,000 bp).

      iv. Corresponding R1 and R2 reads are mapped in either forward-forward or reverse-reverse orientation, instead of forward-reverse orientation or reverse-forward orientation.

      v. One or both have a supplementary split alignment.

      vi. MAPQ of either R1 or R2 is below 17.

      vii. R2 read (barcoded, random fragmented, side) has >3 bp soft clip or <25 bp aligned. It is potentially a chimera read from adapter ligation artifact.

      viii. R1 read (gene specific primer side) has >20 bp soft clip.

5. Gene specific primer identification

   a. Read sequence at the beginning of R1 is compared to the expected primer sequence, using edit distance or Smith-Waterman when edit distance is large.

   b. Discard read pairs for which gene specific primer is not easily identified.

   c. Discard read pairs generated by off-target priming.

   d. Discard read pairs when 5' end of R2 read aligns less than 15 bp from the 3' locus of gene specific primer from corresponding R1 read. Although on-target, this type of read fragment is too short for reliable information in variant calling, e.g. it could be unidentified mis-priming products from gene specific primers.

6. Barcode clustering

   a. Generally a much smaller read family is combined with a much larger read family if their barcodes are within edit distance of 1 and corresponding 5' positions of aligned R2 reads are within 20 bp. More details were described in [1].

   b. New combined/modified barcode information is written into the read ID. An example of final modified read ID is shown below
      `NB500965:27:H7JT7AFXX:2:21112:9631:8678:chr1-1-10291935-GTCACATAATGT:GTCAAATAATGT`. Here, "chr1" represents chromosome 1 where this read sequence is mapped; "-1" represents the reference minus strand which the read sequence matches ("-0" for reference positive strand); "-10291935" represents the mapping position of 5' end of clustered reads; red text string represents the molecular barcode after clustering and blue text string represents the original barcode sequence before clustering. Only the clustered barcodes are used in the downstream analysis.

7. Gene specific primer masking

   a. Modify the .bam file to mask identified primer sequences. Gene specific primer portion at 5' start of in R1 reads, and 3' end of R2 reads will not be used in variant calling. Primer regions are masked using the soft clip CIGAR code.

8. Variant calling using smCounter

   a. smCounter uses the final sorted .bam file as input in variant calling.

   b. The following are default parameters:

  i. minBQ = 20
  ii. minMQ = 30
  iii. hpLen = 10
  iv. mismatchThr = 6.0
  v. mtDrop = 0
  vi. maxMT = 0
  vii. primerDist = 2
  viii. threshold = 0

c. The following read set specific parameters were used on the N0030 dataset. Note that `mtDrop` and `threshold` are not default values.
  i. mtDepth = 3,612
  ii. rpb = 8.6
  iii. mtDrop = 1
  iv. threshold = 60

Parameter settings for MuTect and VarDict

MuTect and VarDict have been implemented to call variants in N0030. Using MuTect, we applied a number of log odds (LOD) scores from 1.0 to 500.0 to create the ROC curve. We turned off the dbSNP filter because our ground truth variant set is in dbSNP and the clustered position filter because DNA fragments have fixed starting positions in amplicon sequencing. For VarDict (Java version), we used different minimum variant allele fractions ranging from 0.2% to 2% to create the ROC curve. Default parameter settings were applied. The MuTect command we used is

```
java -Xmx50g -jar muTect-1.1.4.jar
   --analysis_type MuTect
   --reference_sequence Ref_Genome_hg19.fasta
   --intervals Target_Region.bed
   --input_file:tumor Alignment_File.bam
   --out mutect_extended_stats.out
   --coverage_file mutect_extended.wig.txt
   --vcf mutect_extended.vcf --enable_extended_output
   --fraction_contamination 0.003
   --minimum_mutation_cell_fraction 0.004
   --heavily_clipped_read_fraction 0.75
   --min_qscore 20
   --num_threads 20
   --gap_events_threshold 155
   --downsample_to_coverage 200000
```

The VarDict command we used, assuming the minimum allele fraction is 0.01, is

```
VarDictJava_Path/build/install/VarDict/bin/VarDict \
  -th 20 -F 0 -q 20 -G Ref_Genome_hg19.fasta -f 0.01 -N N0030 -b Alignment_File.bam  \
  -z -c 1 -S 2 -E 3 Target_Region.bed | VarDictJava_Path/VarDict/teststrandbias.R | \
  VarDictJava_Path/VarDict/var2vcf_valid.pl -N N0030 -E -f 0.01 > Variant_Call_Set.vcf
```

Supplementary Figures

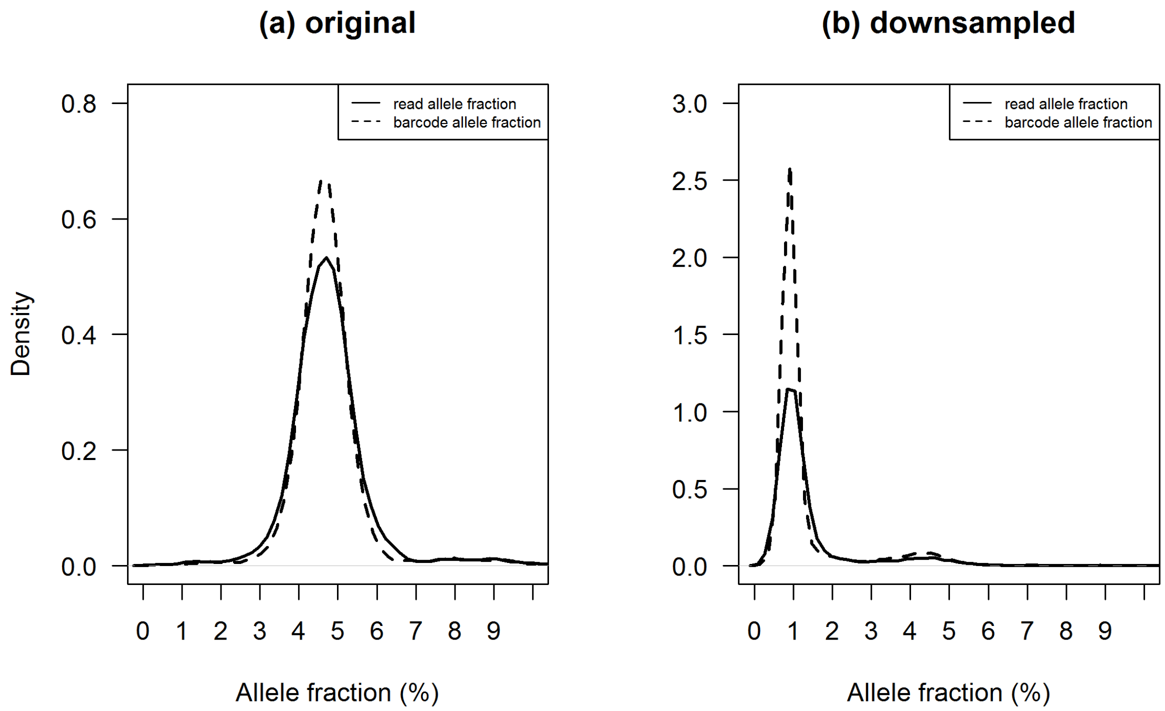

Figure S1: Allele fractions of unique heterozygous NA12878 variants in N0015, in terms of both raw reads and barcodes. (a) Originally the allele fractions were centered around 5%. (b) After downsampling, the allele fractions were centered around 1%, as expected. In both plots, barcode allele fractions are more concentrated around the expected value compared to read allele fraction, because variation from PCR amplification is greatly reduced.

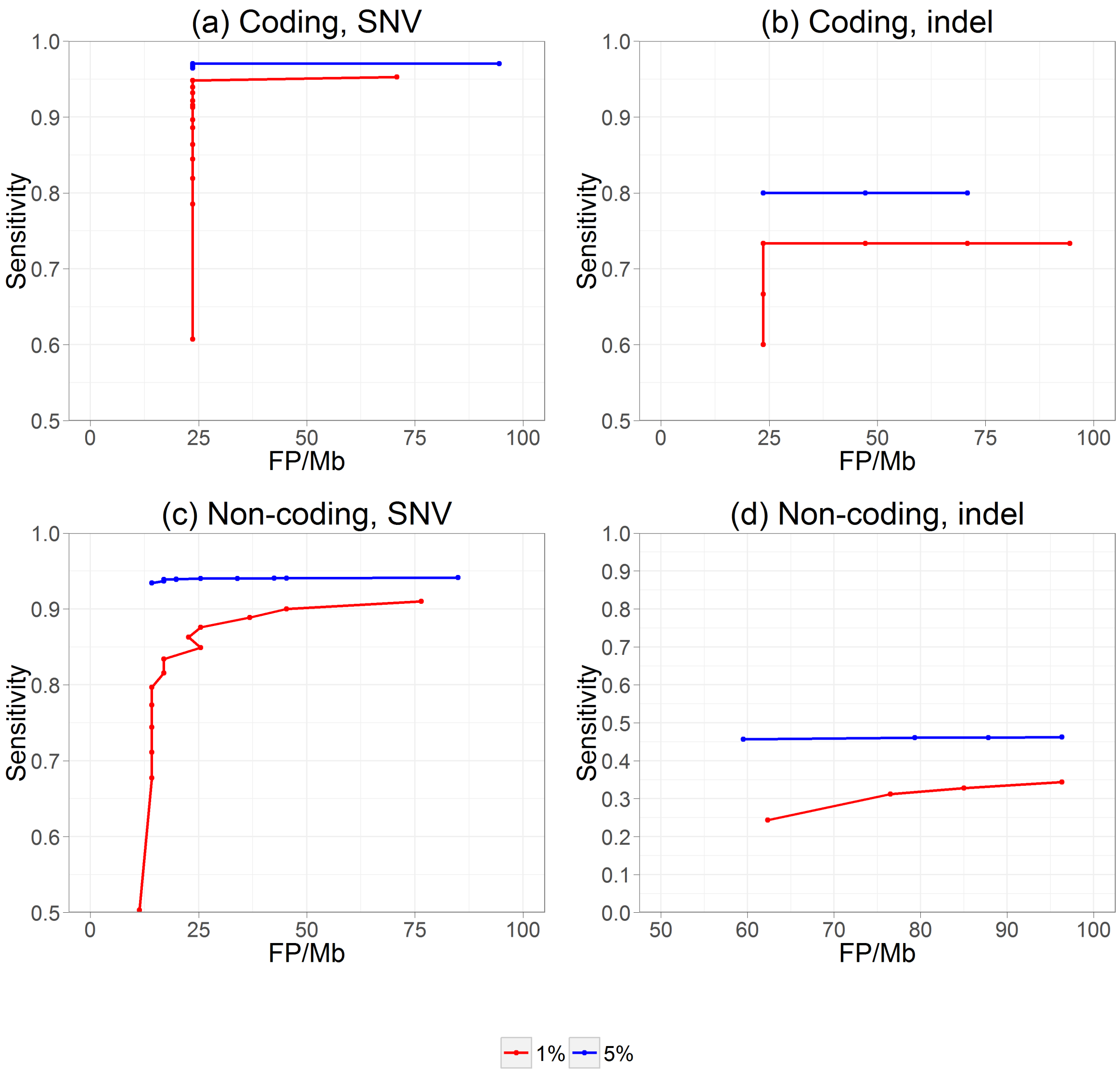

Figure S2: smCounter performance on calling 1% and 5% variants in panel N0015, separated by coding and noncoding regions. X-axis is the number of false positives per megabase and y-axis is sensitivity. Red and blue lines represent 1% and 5% respectively. Each point on the ROC curve represents a threshold value. (a) ROC curves of smCounter on 675 SNVs in coding region. (b) ROC curves of smCounter on 15 indels in coding region. (c) ROC curves of smCounter on 4799 SNVs in non-coding region. (d) ROC curves of smCounter on 686 indels in non-coding region. Note that x and y axis have different limits from the other 3 plots for better visualization.

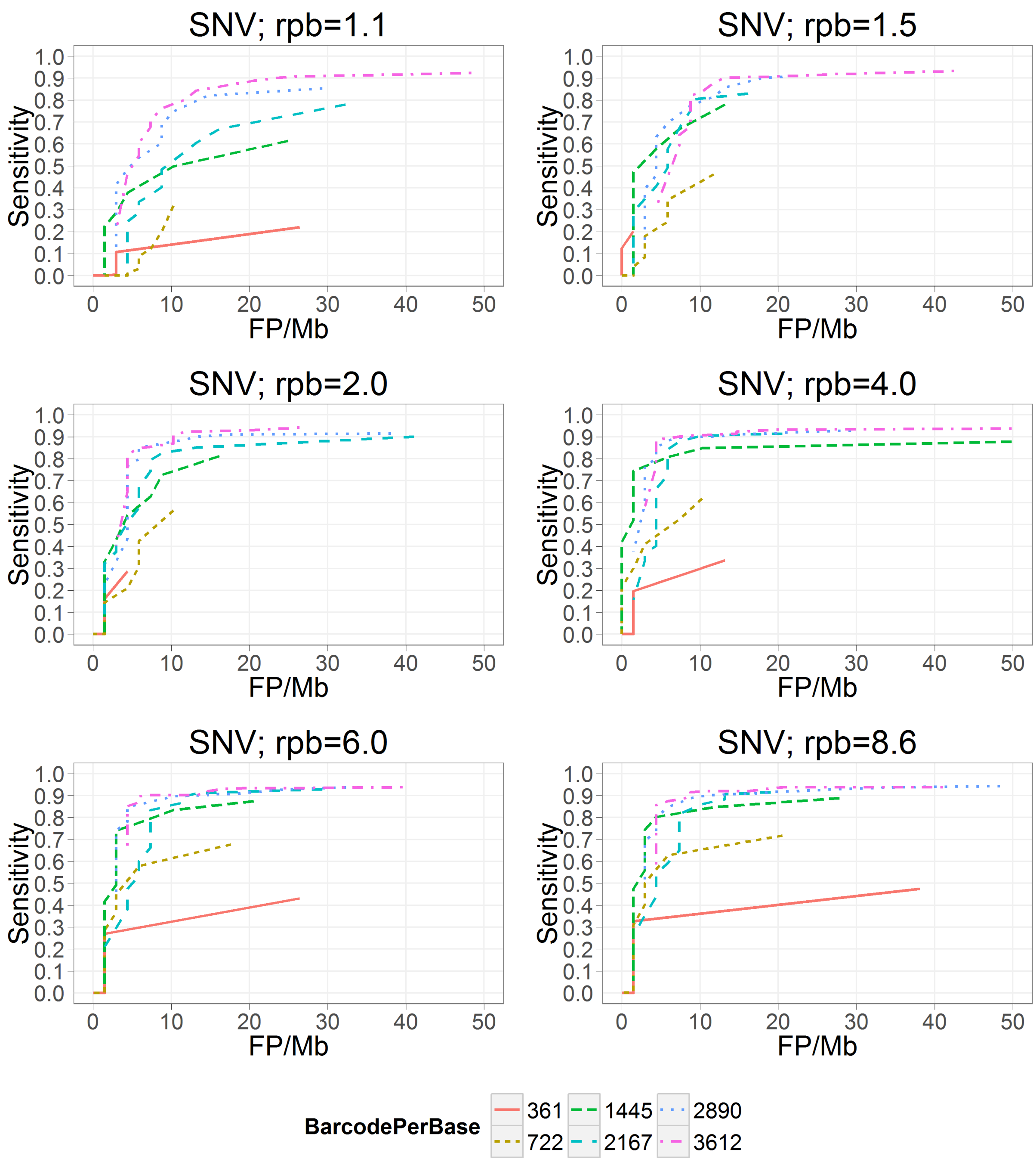

Figure S3: smCounter performance on 223 1% SNVs in N0030 under reduced barcode depth and read pairs per barcode (rpb). Each single plot shows the ROC curves under a fixed rpb and varying barcode depths.

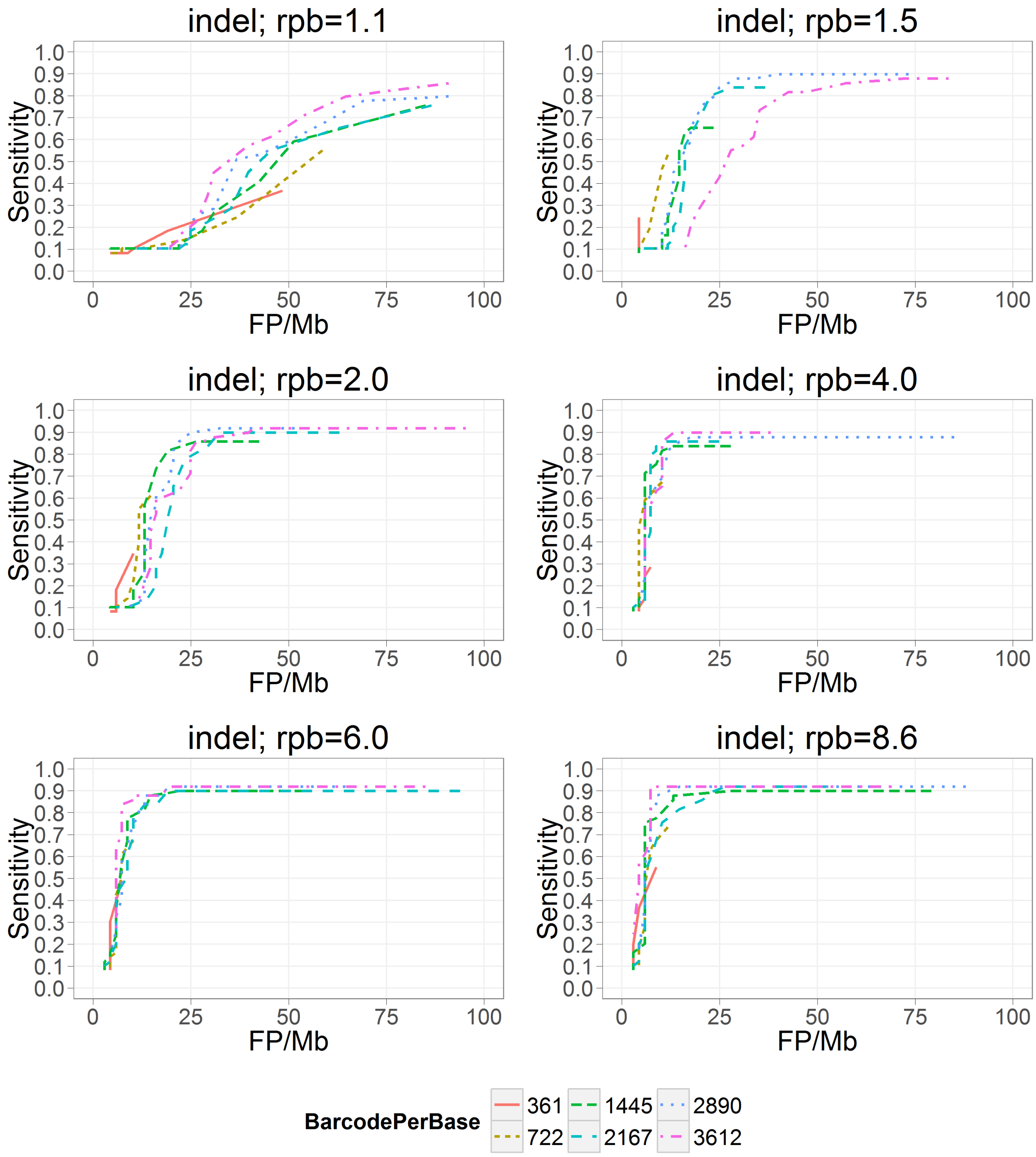

Figure S4: smCounter performance on 49 1% indels in N0030 under reduced barcode depth and read pairs per barcode (rpb). Each single plot shows the ROC curves under a fixed rpb and varying barcode depths.

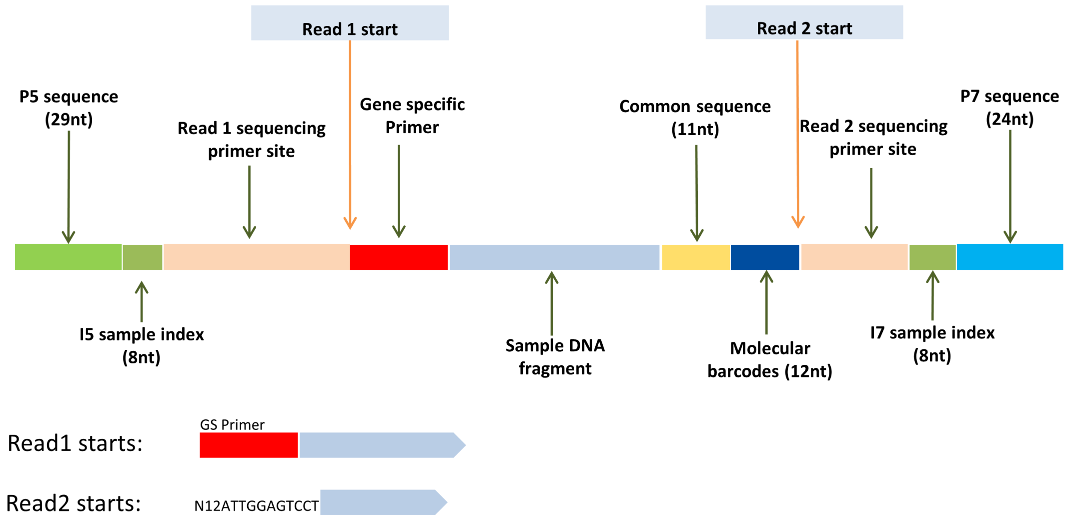

Figure S5: Structure of reads from our enrichment protocol.

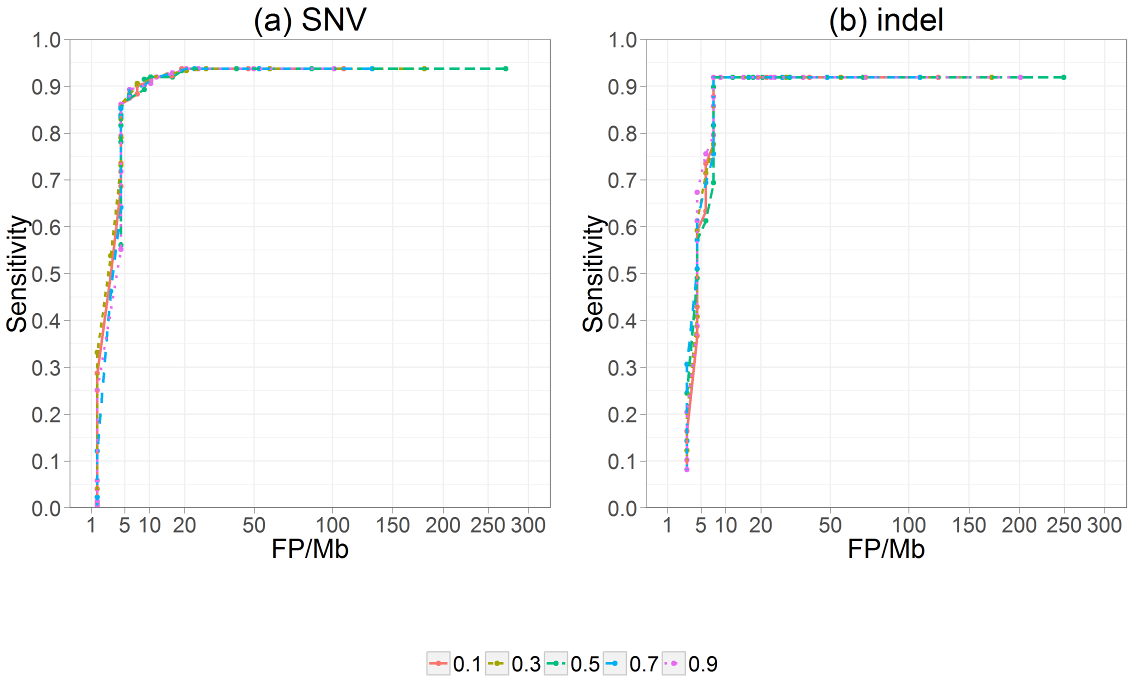

Figure S6: Variant calling performance on 1% variants in panel N0030 with $\alpha = 0.1, 0.3, 0.5, 0.7, 0.9$. Other tested α values give similar ROC curves and are omitted here. (a) ROC curves of smCounter based on 223 SNVs. (b) ROC curves of smCounter based on 49 indels.

Supplementary Tables

Table S1: Performance of smCounter in detecting 5% and *in silico* 1% variants in N0015, separated by coding and noncoding regions. Cutoffs were selected to represent optimal performance.

Panel	Region	Type	TP	FP	FN	TPR(%)	FP/Mb	PPV(%)
N0015(5%)	Coding	SNV	655	1	20	97.0	24	99.8
		indel	12	1	3	80.0	24	92.3
	Non-coding	SNV	4511	9	288	94.0	25	99.8
		indel	323	56	363	47.1	159	85.2
N0015(1%)	Coding	SNV	622	1	53	92.1	24	99.8
		indel	11	2	4	73.0	47	84.6
	Non-coding	SNV	4141	8	658	86.3	23	99.8
		indel	292	56	394	42.6	159	83.9

Table S2: Pre-processing filters and the default settings. The Default settings were applied to obtain the performance data in N0030.

Pre-processing filter	Default setting
Low base quality	Do not use reads at loci with base quality lower than 20
Low map quality	Do not use reads at loci with map quality lower than 30
High mismatch	Discard reads with more than 6 mismatched bases per 100 bp (excluding indels)

Table S3: Post-processing filters and the default settings. Most of the parameters can be adjusted by the user. The default settings were applied to obtain the performance data in N0030.

Post filter	Description	Default parameter settings
Strong barcode	False positives can arise in loci with very high barcode depth even if each barcode only shows weak read evidence. Barcode evidence strength is measured by the barcode-level prediction index (BPI), given by $-\log_{10}\left(1-P(V\|BC_k)\right)$.	Strong barcode is defined as those with BPI < 4.0. Reject variants with less than 2 strong barcodes.
Low quality	High percentage of low quality reads indicates putative false positive.	Reject variant if over 40% of the reads are less than Q20 at the target locus.
Strand bias	Variants observed only or mostly in one strand, possibly due to unbalanced strand mapping [2] or strand specific polymerase errors	Reject variant if odds ratio > 50 or p-value $< 10^{-5}$ in Fisher's exact test.
Homopolymer	False positives caused by misalignment in homopolymer sequences	Reject SNVs within or flanked by 10-base homopolymer sequences.
Simple repeats	False positives caused by misalignment in simple repeat (micro-satellite) region	Reject SNVs within simple repeat sequences in RepeatMasker.
Low complexity	False positives caused by misalignment in low complexity region	In addition to RepeatMasker, low complexity regions also include any 20-base sequence with only 2 types of nucleotides. Variants in these regions are rejected.
End position	Variants observed only near the ends of read caused by ligation or primer artifacts	Reject variants clustered within 20 bases from the random end of the read or 2 bases from the fixed end (downstream of the gene-specific primer).

References

[1] Peng, Q., Satya, R.V., Lewis, M., Randad, P., Wang, Y.: Reducing amplification artifacts in high multiplex amplicon sequencing by using molecular barcodes. BMC Genomics **16**(1), 589 (2015)

[2] Guo, Y., Li, J., Li, C.-I., Long, J., Samuels, D.C., Shyr, Y.: The effect of strand bias in illumina short-read sequencing data. BMC Genomics **13**(1), 1–11 (2012). doi:10.1186/1471-2164-13-666
